# Malaria risk stratification in Lao PDR guides program planning in an elimination setting

**DOI:** 10.1038/s41598-024-52115-2

**Published:** 2024-01-19

**Authors:** Phoutnalong Vilay, Julia C. Dunn, Odai Sichanthongthip, Rita Reyburn, Phonephet Butphomvihane, Vilaisak Phiphakavong, Punam Amratia, Mary Hahm, Vilayphone Phongchantha, Chitsavang Chanthavisouk, Boualam Khamlome, Keobouphaphone Chindavongsa, Virasack Banouvong, Matthew Shortus

**Affiliations:** 1 Center of Malariology, Parasitology and Entomology, Vientiane, Lao PDR; 2 Clinton Health Access Initiative, Vientiane, Lao PDR; 3 World Health Organization, Vientiane, Lao PDR; 4 Malaria Atlas Project, Telethon Kids Institute, Perth, Australia

**Keywords:** Epidemiology, Malaria

## Abstract

Malaria in Lao People’s Democratic Republic (Lao PDR) has declined rapidly over the last two decades, from 279,903 to 3926 (99%) cases between 2001 and 2021. Elimination of human malaria is an achievable goal and limited resources need to be targeted at remaining hotspots of transmission. In 2022, the Center of Malariology, Parasitology and Entomology (CMPE) conducted an epidemiological stratification exercise to assign districts and health facility catchment areas (HFCAs) in Lao PDR based on malaria risk. The stratification used reported malaria case numbers from 2019 to 2021, risk maps derived from predictive modelling, and feedback from malaria staff nationwide. Of 148 districts, 14 were deemed as burden reduction (high risk) districts and the remaining 134 as elimination (low risk) districts. Out of 1235 HFCAs, 88 (7%) were classified as highest risk, an improvement from 187 (15%) in the last stratification in 2019. Using the HFCA-level stratification, the updated stratification resulted in the at-risk population (total population in Strata 2, 3 and 4 HFCAs) declining from 3,210,191 to 2,366,068, a 26% decrease. CMPE are using the stratification results to strengthen targeting of resources. Updating national stratifications is a necessary exercise to assess progress in malaria control, reassign interventions to the highest risk populations in the country and ensure greatest impact of limited resources.

## Introduction

The Lao People’s Democratic Republic (Lao PDR) aims to eliminate all human species of malaria by 2030^1^. Between 2001 and 2021 reported malaria cases have declined from 279,903 to 3926, a 99% decline^2,3^. In 2021, 35% of reported cases were *Plasmodium falciparum* or mixed infections with the majority being *P. vivax* infections. Malaria deaths are rare; only one death due to malaria was reported between 2019 and 2021^4^.

**Figure 1:**
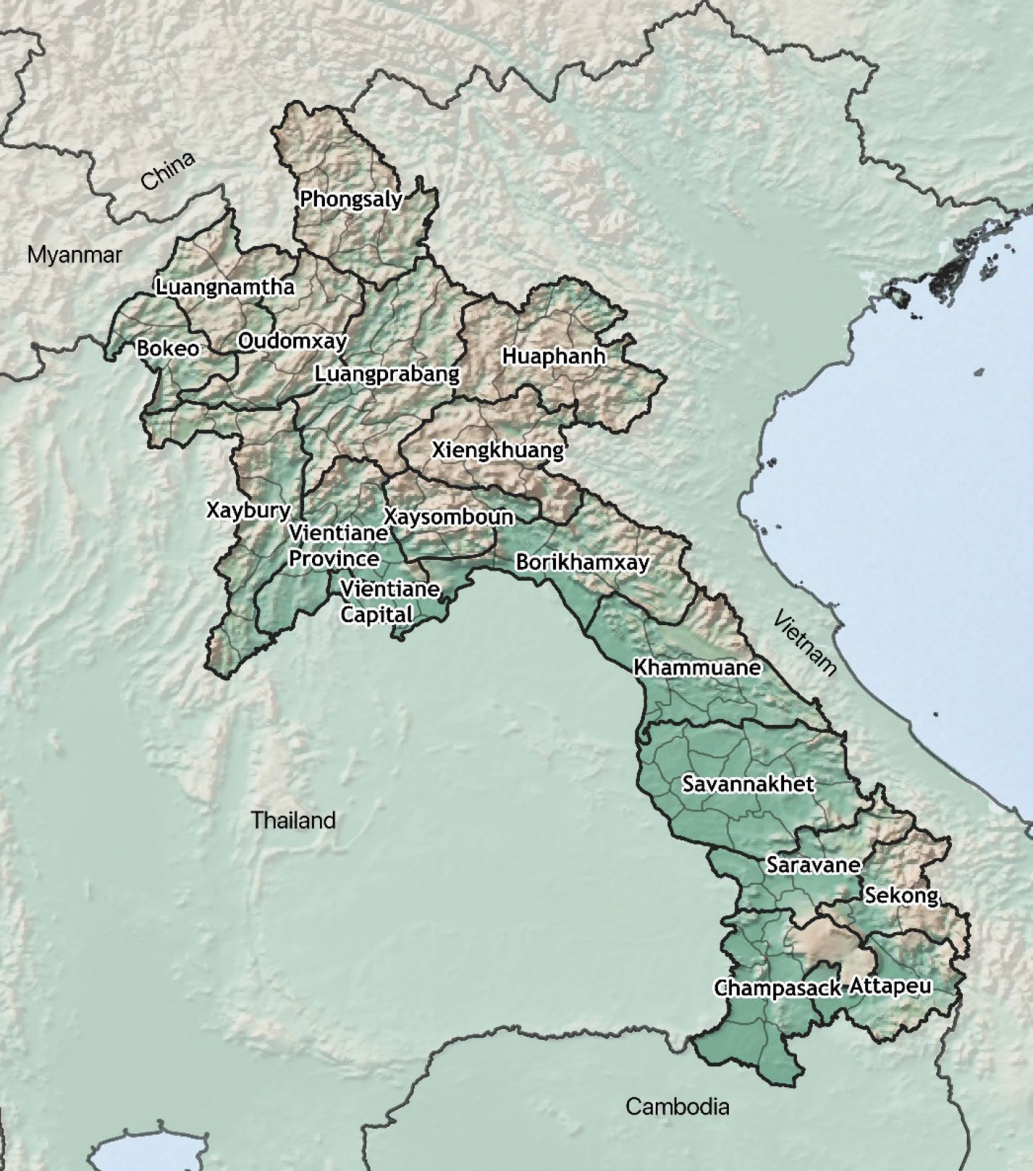
Provinces of the Lao People’s Democratic Republic. Map created using QGIS, version 3.34.1 (http://www.qgis.org/)^6^.

In 2020, the Center of Malariology, Parasitology and Entomology (CMPE) in Lao PDR published the National Strategic Plan (NSP) for malaria control and elimination 2021–2025^5^. The strategy aims to reduce annual parasite incidence per 1000 population (API) to below 0.16 by 2025, with an intermediate target of 0.55 by 2021, which was achieved through strengthening the surveillance systems, distributing long lasting insecticidal nets (LLINs) to high-risk populations, expanding testing and improving case management. Figure 1 presents the provinces of Lao PDR. Out of 18 provinces, only two had an API above one in 2021: Sekong (5.9) and Attapeu (9.3). Malaria in Lao PDR is becoming increasingly focalised. In 2018, 80% of cases were reported from 67 health facilities (5%). In 2021 this had decreased to 30 health facilities (2%)^3^. A key component of the NSP is continued use of malaria data to identify areas with consistent malaria transmission as malaria declines and to ensure that control efforts are intensified in these areas.

Malaria risk stratification is recommended in the World Health Organization (WHO) framework for malaria elimination^7^ and has been applied in high burden malaria endemic countries such as Mali^8^ and Colombia^9^, and lower burden Greater Mekong Subregion (GMS) countries such as Cambodia^10^ and Thailand^11^. CMPE first conducted a malaria risk stratification for Vientiane Province in 1990^12^. Subsequent stratifications were completed in 1997^13^, 2008^14,15^, 2012, 2016^16^ and 2019^5^. The most recent stratification in 2019 was conducted at district level and HFCA level and was used to inform the NSP, calculate case and testing targets, design national surveillance and response strategy, and allocate village malaria workers (VMWs) and LLINs to high-risk villages.

In early 2022, CMPE, with the support of the WHO, the Clinton Health Access Initiative (CHAI) and modelling outputs from the Malaria Atlas Project (MAP), updated the stratification to inform strategic planning and costing for the Regional Artemisinin-resistance Initiative (RAI4E) based on the most up-to-date understanding of malaria epidemiology in the country. CMPE conducts epidemiological stratification at district level and HFCA level. This two-tiered approach allows CMPE to inform strategy decisions tailored to the appropriate implementation level. District stratifications inform broader policy implementation such as surveillance processes e.g. introduction of case investigation, classification and foci response, whereas HFCA stratification informs allocation of resources e.g. VMWs and long-lasting insecticide treated nets (LLINs). The aim of the 2022 stratification was to refocus malaria elimination efforts on the remaining hotspots of transmission in Lao PDR, to inform elimination strategy and support quantification of malaria commodities.

## Methods

In January 2022 the stratification technical working group (TWG) was formed comprising of members from CMPE, WHO, CHAI and the United Nations Office for Project Services (UNOPS). Roles and responsibilities were assigned to members and a completion date of April 2022 was set.

### Data collation and cleaning

The stratification was based on a combination of passively and actively collected data from all health providers in Lao PDR. The main data source for the stratification was the routine malaria surveillance data collated in Lao PDR’s health management information system, hosted on District Health Information Software 2 (DHIS2)^3^. Lao PDR’s malaria DHIS2 collects both case-based and HF-level aggregate data. According to Lao PDR’s surveillance guidelines, all confirmed malaria cases should be reported into DHIS2. Aggregate data on the number of malaria tests is also available. At the time of the stratification exercise, case investigation and classification were not conducted for cases reported in burden reduction districts (87.9% of the cases in the stratification analysis) and completeness was low in elimination districts. As such, the majority of reported cases included in the stratification analysis did not have a local/imported classification and did not have travel history information. Therefore, this data was not included in the stratification. For the district-level stratification, annual total reported confirmed malaria cases and total population from 2019 to 2021 for every district was extracted from DHIS2. For the HFCA-level stratification, annual data (total reported malaria cases, total number of tests conducted and reporting completeness) from 2019 to 2021 was extracted from DHIS2 for every public health facility, including data from VMWs managed through that health facility, in Lao PDR.

Additional data cleaning was conducted on data from district hospitals, province hospitals, private facilities and army hospitals. These facilities are often preferred for medical consultation due to the perceived higher standard of care or urban location which results in cases travelling longer distances to these facilities while residing in a location outside of the defined population catchment area—creating a bias in case reports at higher-level facilities. To resolve this issue the TWG decided to assign all cases that reported at these facilities to the HFCA of their residential address. The residential villages of cases were mapped, and each case was reassigned to the geographically closest primary health facility. This exercise resulted in reassignment of 4556 cases (32.6% of the total cases included in the analysis); 3486 cases reporting to a district or provincial hospital, 804 cases reporting to a private health facility, and 266 cases reporting to an army hospital. This exercise was not conducted on data from health centers (HCs) as it was assumed that cases reported at HCs were from villages within the health facility catchment area, therefore reassignment was unnecessary. The same method was applied to cases that were detected via active case detection (ACD) during outbreak response, as these cases are reported in DHIS2 under the district health office instead of the nearest HF, of which there were 1031 cases (7.4% of the total cases included in the analysis).

### Stratification methods

#### District level

For the district level stratification, the same method was used as in 2019. The three-year API per 1000 population was calculated for each district. Following the thresholds set out in the Lao PDR NSP^5^, districts with an API under one were designated as “elimination districts”, districts with an API equal to or above one were designated as “burden reduction” districts. Results were discussed with central and province level malaria staff to ensure the appropriateness of the stratifications on an operational level.

#### Health facility catchment area (HFCA) level

The 2022 stratification was conducted at HFCA level as the majority of malaria interventions in Lao PDR are implemented by health facilities (Table 1). For reference, there is an average of 5.8 villages (range 1–28) per HFCA according to the Laos Health Facility Master List. As described above, data from private facilities and army hospitals were included but the facilities themselves were not included in the stratification analysis and did not receive a strata designation. District and province hospitals were included in the stratification analysis and received a strata designation. A total of 1235 HFCAs were included in the stratification analysis. The HFCA-level stratification consisted of four stages: (1) epidemiological stratification based on reported cases, (2) risk map development, (3) consolidation of strata based on cases and the risk map, and (4) operational considerations.

**Table 1:** Intervention packages targeted to each strata.

Strata	Intervention packages
District-level stratification	
Elimination	Surveillance for elimination—case notification, case investigation, case classification, foci investigation, foci response
Burden reduction	Surveillance for control and effective response in areas with increased transmission
HFCA-level stratification	
Malaria free	1. Universal testing and treatment 2. IEC
Stratum 2	1. Universal testing and treatment 2. IEC
Stratum 3	1. Universal testing and treatment 2. IEC 3. Targeted LLIN coverage 4. Targeted VMWs (villages with at-risk populations) 5. Outbreak or foci response (ACD, IRS, entomological surveillance, LLIN top-up, targeted drug administration (TDA) in areas with prolonged outbreaks)
Stratum 4	1. Universal testing and treatment 2. IEC 3. Universal LLIN coverage 4. Universal VMWs 5. Outbreak or foci response (ACD, IRS, entomological surveillance, LLIN top-up, targeted drug administration in areas with prolonged outbreaks) 6. Targeted Mobile Malaria Workers (for high-risk populations staying outside of the village) 7. TDA and intermittent preventative treatment (for high-risk populations during malaria high season in targeted villages)

*HFCA* health facility catchment area, *IEC* information, education, communication, *LLIN* long-lasting insecticide treated net, *VMW* village malaria workers, *ACD* active case detection, *IRS* indoor residual spraying, *TDA* targeted drug administration.

After a consultation between CMPE, WHO and partners, the same methods were used in the 2019 and 2022 stratifications aside from three key differences, which are described in more detail throughout the methods. These key differences were (1) 27 HFCAs included in the 2022 stratification did not have strata assigned in 2019. This includes central level hospitals and health facilities that have newly opened since 2019, (2) reassigning cases from district hospitals, province hospitals, private facilities and army hospitals to the HFCA of the patient’s residential address, (3) reassignment of cases detected via ACD during outbreak response. As such, the results of the stratifications can be compared to each other to analyse progress in malaria elimination in Lao PDR.

##### Epidemiological stratification based on reported cases

For the HFCA stratification, whole case numbers were used instead of API, as malaria burden in Lao PDR is now low enough that it is feasible to target all areas reporting cases to achieve elimination. Total cases were used, instead of considering *P. falciparum* and *P. vivax* separately, because the malaria interventions informed by the stratification are the same regardless of malaria species. Commodity forecasting for radical cure, which is available for *P. vivax* treatment nationwide, is not informed by the stratification. For the first stage of HFCA level stratification, HFCAs were assigned to one of four strata denoting malaria risk based on the total reported malaria cases from 2019 to 2021. HFCAs with zero cases were classed as “Malaria free”, 1–4 cases were “Stratum 2”, 5–20 cases were “Stratum 3”, more than 20 cases were “Stratum 4”. The case thresholds to define each strata were defined by the TWG during the 2019 stratification exercise. Based on the serial interval between successive cases (period from the gametocyte stage of the primary case to the earliest appearance of gametocyte in the secondary case) of malaria typically between 25 and 50 days (depending on species)^17,18^ it was decided that catchments with less than 5 cases within the assessment period (32 months) were likely to be mainly imported cases, whereas catchments with between 5 and 20 were likely to be a mixture of imported cases and locally acquired cases. Catchments with over 20 cases were highly likely to have had some level of active transmission during the 32-month period so were considered the highest risk. However, any HFCAs with fewer than 10 tests or below 50% average reporting completeness over the three years were deemed to have unreliable data and were classed as “Unknown” to be assigned a stratum in the following stages.

##### Risk map development

To validate the results from the first stage of the stratification and estimate vulnerability in the “Unknown” HFCAs, a predictive risk model was developed by MAP. A complete description of the methods is included in Kang and Amratia et al*.* 2023 (*In Preparation*). In brief, a predictive geostatistical model, developed by MAP, was created that relies on information about reported malaria cases and considers various factors like the environment and social characteristics of the area. The goal was to predict the number of malaria cases in these regions between 2019 and 2021.

A geostatistical framework was used to allow to interpolation of risk to unknown locations. For this the model was built in two-fold. First, a likelihood was chosen to describe the response e.g. malaria case counts. A negative binomial likelihood was chosen given the large dispersion of zero case counts. The likelihood is fit in a regression style and includes a layer with an intercept and various factors describing the environment and socio-demographics, and a random field to account for the spatial relationship between different places. The model also considered 16 different factors—these include access to cities^19^, aridity^20^, distance to water^21^, elevation^22^, night-time lights^23^, potential evapotranspiration^20^, slope^22^, temperature suitability index^24^, tree fraction^25^, rainfall^26^, enhanced vegetation index^27^, day and night land surface temperature^28^, tasselled cap brightness^29^, tasselled cap wetness^29^. For more details these covariates are described in *Kang and Amratia *et al*. 2023 (In Preparation)*. Second, to fit the correct denominator for malaria case counts in health facilities a catchment model was also used to estimate the catchment population per health facility. The catchment model was designed on a modified gravity-style approach, which takes into account travel time. Unlike a traditional model, this approach allows for overlapping catchment areas between health facilities, reflecting how people might seek care from multiple locations rather than just one. The model also considers the attractiveness of a health facility, taking into account factors like facility type, ownership, and available services. It was assumed that an individual would not visit more than four facilities and would not travel more than 3 h for care or treatment.

##### Consolidation of strata based on cases and the risk map

Each HFCA with latitudes and longitudes were all assigned a secondary stratification based entirely on the total estimated malaria cases calculated from the risk map and using the same thresholds described above. Any HFCAs deemed “Unknown” and had latitudes and longitudes available were assigned this stratum. In situations where a HFCA’s malaria burden was unknown and coordinates were missing, meaning that estimated malaria case totals could not be extracted from the risk map, the mean number of HFCA cases for the entire district was used. All other HFCAs had their first stratification (from reported cases) and second stratification (from modelled estimated cases) compared. All HFCAs with major discrepancies between the two strata were discussed one by one by the TWG. The TWG then assigned a stratum based on reported cases, estimated cases, strata of surrounding HFCAs, and knowledge of historical and existing interventions in those areas. For example, if an HFCA with existing VMWs was flagged to move into a stratum where the VMWs would be removed, the malaria data and HFCA context were discussed to consider whether there was ongoing risk of transmission in that area and whether removing the VMWs may risk resurgence.

##### Operational considerations

To ensure that stratification results were informed by the extensive knowledge and experience of the malaria control staff in Lao PDR and not just by reported data which may have unmeasured biases, results of the district and HFCA level stratifications were shared with provincial and district anti-malaria stations. Feedback on the appropriateness of the stratifications was consolidated through online meetings where provincial and district staff gave feedback based on their in-depth knowledge of the population dynamics, risk behaviors and risk locations for their area. Their feedback was considered when finalising the stratification results.

### Ethics approval and consent to participate

The requirement for ethical approval was waived by Dr Virasack Banouvong, Director of the Center for Malaria, Parasitology and Entomology (CMPE), granted authority to do so by the National Ethics Committee for Health Research (NECHR). The requirement for informed consent was also waived by Dr Virasack Banouvong, Director of CMPE, granted authority to do so by the NECHR, as human data was collected via routine disease surveillance, aggregated and anonymized. All methods were carried out in accordance with NECHR guidelines and regulations.

## Results

### District level

Based on district-level API, 15 of 148 districts were classified as burden reduction districts and 133 were classified as elimination districts. One adjustment was made; Boualapha district in Khammuane Province was not classified as a burden reduction district despite its API of 2.66. As Boualapha was classified as an elimination district in the 2019 stratification, it was decided in the province validation phase to keep Boualapha as an elimination district to avoid removing elimination activities that had already begun. After this adjustment, there were 14 burden reduction districts and 134 elimination districts. This is an improvement from 2019 where 23 districts were classified as burden reduction. A summary of the district-level stratification results are presented by province in Table 2.

**Table 2:** District level stratification results from the 2019 and 2022 stratifications.

Province	Districts	Districts in elimination phase (API < 1) (%)		Districts in burden reduction phase (API ≥ 1) (%)	
		2019	2022	2019	2022
Vientiane Capital	9	9 (100)	9 (100)	0	0
Phongsaly	7	7 (100)	7 (100)	0	0
Luangnamtha	5	5 (100)	5 (100)	0	0
Oudomxay	7	7 (100)	7 (100)	0	0
Bokeo	5	5 (100)	5 (100)	0	0
Luangprabang	12	12 (100)	12 (100)	0	0
Huaphanh	10	10 (100)	10 (100)	0	0
Xaybury	11	11 (100)	11 (100)	0	0
Xiengkhuang	7	7 (100)	7 (100)	0	0
Vientiane Province	11	11 (100)	11 (100)	0	0
Borikhamxay	7	7 (100)	7 (100)	0	0
Khammuane	10	10 (100)	10 (100)	0	0
Savannakhet	15	11 (73)	13 (87)	4 (27)	2 (13)
Saravane	8	1 (13)	5 (63)	7 (88)	3 (37)
Sekong	4	1 (25)	1 (25)	3 (75)	3 (75)
Champasack	10	6 (60)	9 (90)	4 (40)	1 (10)
Attapeu	5	0	0	5 (100)	5 (100)
Xaysomboun	5	5 (100)	5 (100)	0	0
Lao PDR	148	125 (84)	134 (91)	23 (16)	14 (9)

### Health facility catchment area (HFCA) level

Table 3 presents the stages of assigning strata to the 1235 HFCAs included in the Lao PDR 2022 stratification based on reported cases and the risk map (stages 1 and 3 detailed in the “Methods”). From assessing testing and reporting data, 68 HFCAs (6%) were initially deemed to have unreliable data and were assigned as “Unknown”. These HFCAs were stratified using modelled case estimates, district-level data and CMPE discussions: 56 as malaria-free (82% of the 68 “Unknown” HFCAs), six as Stratum 2 (9% of the “Unknown” HFCAs), six as Stratum 3 (9% of the “Unknown” HFCAs) and zero as Stratum 4.

**Table 3:** Changes in health facility catchment area (HFCA) strata through the stages of stratification analysis.

Steps	Stratum 1 (malaria free)	Stratum 2 (low risk)	Stratum 3 (medium risk)	Stratum 4 (high risk)	Unknown
Step 1: Classify by reported case count	849	155	75	88	68
Step 2: Assign unknown HFCAs with coordinates using modelled estimated case totals	877	167	80	92	19
Step 3: Assign unknown HFCAs without coordinates using district level case average	894	167	82	92	0
Step 4: Reassign HFCAs with discrepant reported/estimated case strata	857	193	97	88	0

**Figure 2:**
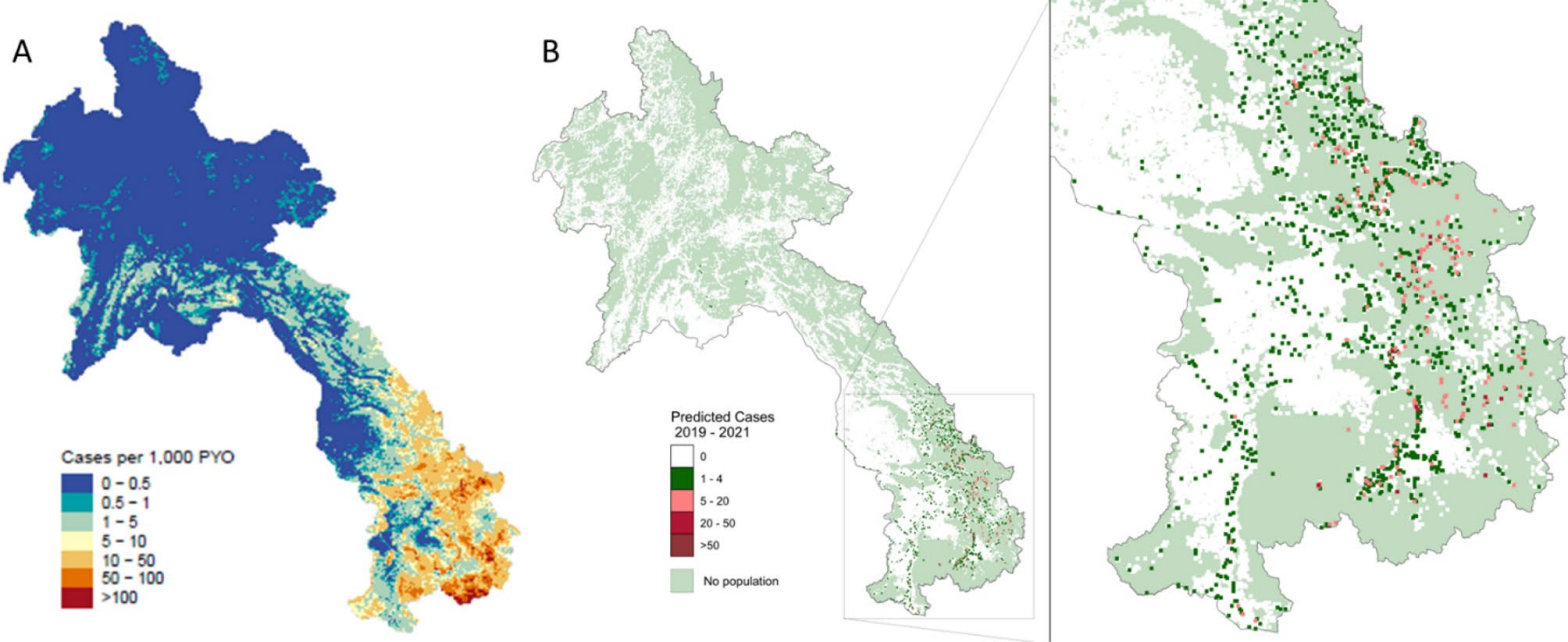
Fine scale maps of (**A**) estimated median of annual malaria cases per 1000 person-years-observed (PYO) in Lao PDR for 2019–2021. API < 1 case per 1000 population is used to define elimination vs burden reduction regions (source: Kang and Amratia et al. 2023). (**B**) represents the annual average of predicted cases between 2019 and 2021 generated by MAP with inset focusing on higher transmission zone in the south of the country. Pixels are visualised at 2km resolution. Areas in light green are where no population exist (source: Laos HRSL population, 2020^30^). Maps created using R, version 4.2.1 (https://www.R-project.org/)^31^.

**Figure 3:**
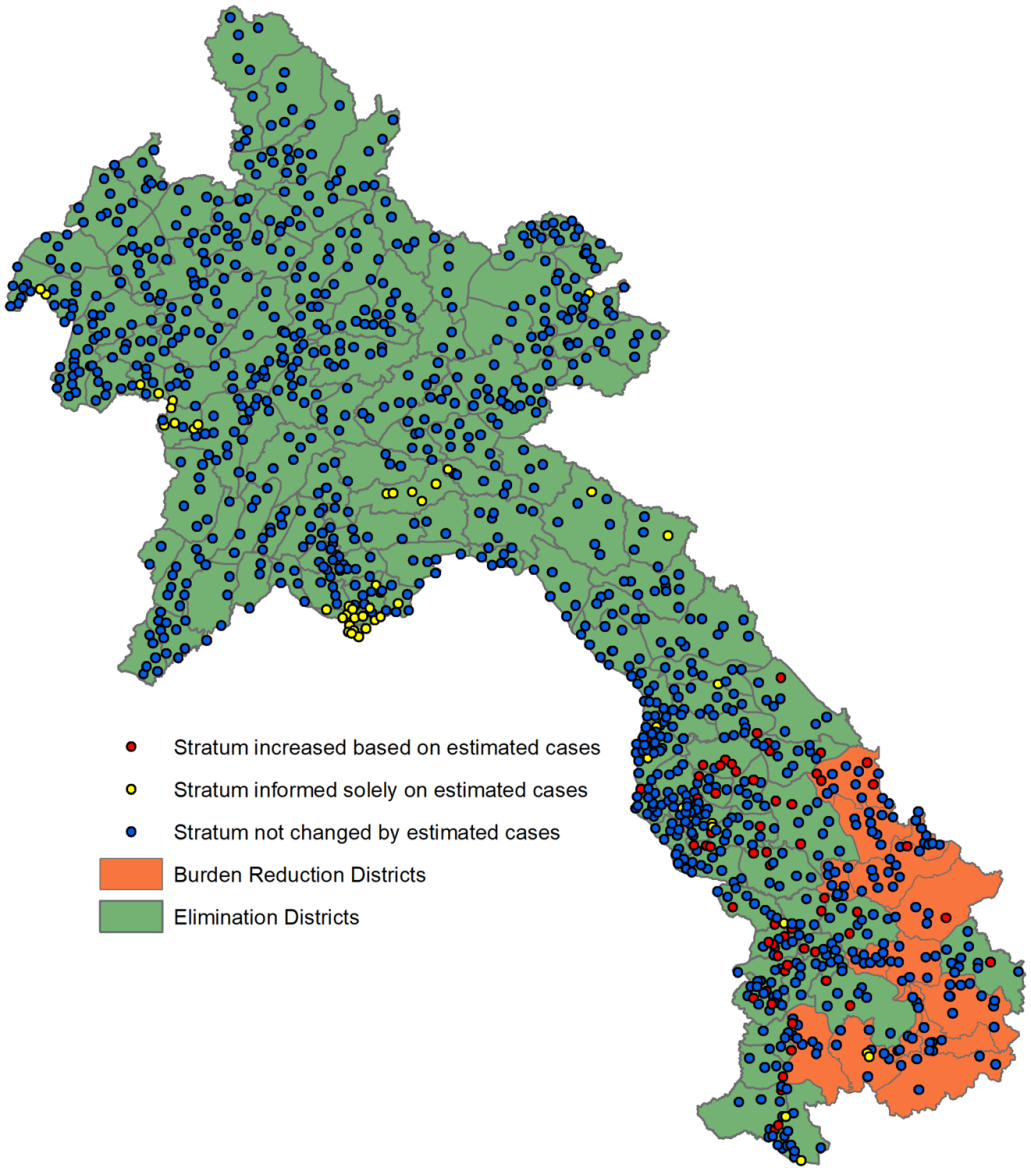
The change in health facility catchment area (HFCA) strata from validation against estimated case totals. Excluding 77 HFCAs without geographic coordinates. Map created using ArcGIS, version 10.7.1 (https://www.arcgis.com/index.html)^32^.

The step of validating strata assigned from reported case data vs strata assigned from modelled case estimates resulted in 62 HFCAs being moved to higher risk strata. Figure 2 shows the risk map generated by MAP and Fig. 3 shows the HFCAs with strata changed by the risk map outputs. The majority (50, 81%) had originally been classified as Malaria Free but the risk map estimated higher underlying risk of transmission. As such, to avoid removing interventions too early and risking resurgence, 45 of these HFCAs had their strata increased to Stratum 2 and five to Stratum 3. No HFCA level stratifications were altered from discussions with the provincial and district anti-malaria stations. There is a cluster of HFCA strata informed solely by estimated cases in central hospitals in Vientiane Capital province. This is due to a unique situation where any malaria tests done in these HFs are sent to laboratories and reported by them into DHIS2, instead of through the hospital, resulting in the hospital itself being flagged for poor testing rates and therefore relying on the estimated case data for the stratification. Other clusters are in Xaisathan district, Xaybury province and Anouvong district, Xaysomboun province. These are due to being assigned as “Unknown” due to poor testing and reporting rates. These health facilities require further supervision to ensure their testing and reporting is improved. All were assigned as Malaria Free, which is the same stratum they would have been assigned based on reported data alone.

**Figure 4:**
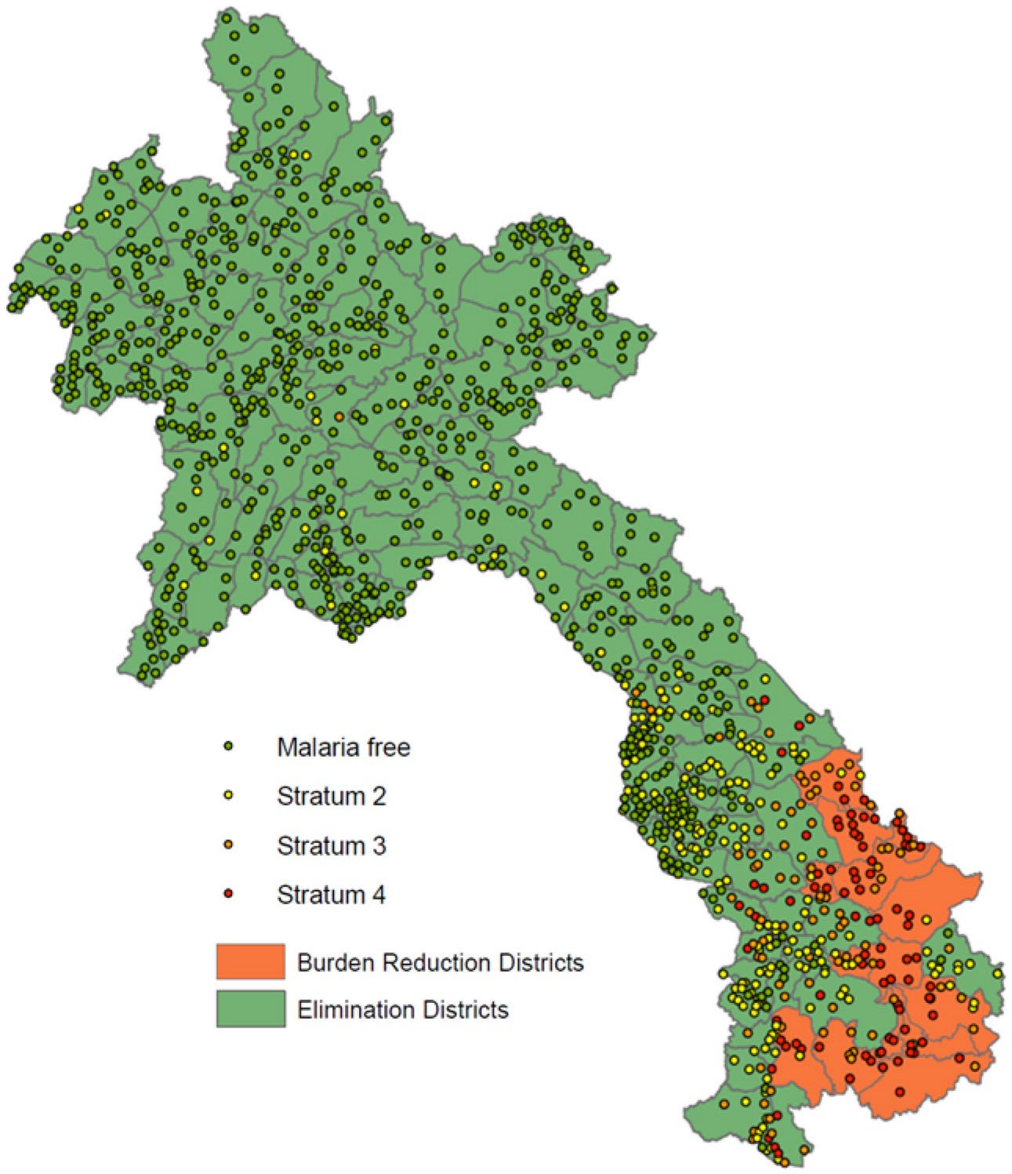
2022 stratification results at district and health facility catchment area level, and aggregated by province. The map excludes 77 HFCAs without available latitude and longitude data. Map created using ArcGIS, version 10.7.1 (https://www.arcgis.com/index.html)^32^.

Figure 4 maps the district-level stratifications and the HFCA-level stratifications for all HFCAs with geographic coordinates. All Strata 3 and 4 HFCAs, except one in Luangprabang province, are located in the six southernmost provinces. Of the 88 Stratum 4 HFCAs, 70 (80%) are in burden reduction districts and 18 (20%) are in elimination districts. Of the 97 Stratum 3 HFCAs, 39 (40%) are in burden reduction districts and 58 (60%) are in elimination districts.

Table 4 presents the results of the 2022 stratification by province, compared to the 2019 stratification results. The number of HFCAs in the highest risk stratum, Stratum 4, declined from 187 in 2019 to 88 in 2022 (53%) and Malaria Free HFCAs increased from 740 in 2019 to 857 in 2022 (14%) clearly showing the decline in malaria cases in Lao PDR over the last three years.

**Table 4:** Number and % of health facilities in each strata from the 2019 and 2022 stratifications.

Province	Stratum 1 (malaria free)		Stratum 2 (low risk)		Stratum 3 (medium risk)		Stratum 4 (high risk)	
	2019	2022	2019	2022	2019	2022	2019	2022
Vientiane Capital	30 (71)	49 (98)	11 (26)	1 (2)	1 (2)	0	0	0
Phongsaly	21 (40)	54 (95)	24 (46)	3 (5)	3 (6)	0	4 (8)	0
Luangnamtha	39 (89)	43 (96)	4 (9)	2 (4)	1 (2)	0	0	0
Oudomxay	52 (87)	60 (100)	7 (12)	0	1 (2)	0	0	0
Bokeo	45 (98)	46 (100)	1 (2)	0	0	0	0	0
Luangprabang	77 (82)	92 (96)	13 (14)	3 (3)	4 (4)	1 (1)	0	0
Huaphanh	84 (99)	84 (99)	1 (1)	1 (1)	0	0	0	0
Xaybury	76 (87)	84 (95)	9 (10)	4 (5)	2 (2)	0	0	0
Xiengkhuang	59 (95)	61 (97)	3 (5)	2 (3)	0	0	0	0
Vientiane Province	46 (81)	55 (95)	9 (16)	3 (5)	2 (4)	0	0	0
Borikhamxay	32 (65)	44 (90)	16 (33)	5 (10)	1 (2)	0	0	0
Khammuane	70 (70)	69 (68)	19 (19)	23 (23)	5 (5)	7 (7)	6 (6)	2 (2)
Savannakhet	71 (41)	74 (42)	41 (24)	62 (35)	24 (14)	23 (13)	36 (21)	16 (9)
Saravane	8 (10)	8 (10)	7 (9)	22 (28)	16 (20)	28 (35)	49 (61)	22 (28)
Sekong	6 (19)	2 (6)	4 (13)	12 (35)	6 (19)	7 (21)	15 (48)	13 (38)
Champasack	8 (9)	12 (14)	12 (14)	43 (49)	18 (21)	19 (22)	49 (56)	13 (15)
Attapeu	0	0	1 (3)	4 (11)	10 (26)	12 (32)	27 (71)	22 (58)
Xaysomboun	16 (73)	20 (87)	5 (23)	3 (13)	0	0	1 (5)	0
Lao PDR^a^	740 (61)	857 (69)	187 (15)	193 (16)	94 (8)	97 (8)	187 (15)	88 (7)

^a^27 HFCAs included in the 2022 stratification did not have strata assigned in 2019. This includes central level hospitals and health facilities that have newly opened since 2019.

Table 5 shows the number of HFCAs in each strata in 2019 and 2022 as well as the total populations in each strata using the 2022 HFCA population at-risk with the aim of presenting how updating the national stratification also changes the total population at-risk. CMPE defines the at-risk population as the total population in Strata 2, 3 and 4 HFCAs. Between 2019 and 2022, the at-risk population declined from 3,210,191 to 2,366,068, a 26% decrease. If the stratification had not been updated, an additional 90 HFCAs and 844,123 people would continue to be targeted for higher intensity interventions.

**Table 5:** Comparison of HFCA strata and population from 2019 and 2022 stratifications.

Strata	HFCA results of 2019 Stratification		HFCA results of 2022 Stratification	
	n HFCA (%)	Population in these strata using 2022 population estimates (%)	n HFCA (%)	Population in these strata using 2022 population estimates (%)
Stratum 1 (malaria free)	740 (61)	4,152,174 (56)	857 (69)	5,034,669 (68)
Stratum 2 (low risk)	187 (15)	1,464,610 (20)	193 (16)	1,243,685 (17)
Stratum 3 (medium risk)	94 (8)	546,866 (7)	97 (8)	638,777 (9)
Stratum 4 (high risk)	187 (15)	1,198,715 (16)	88 (7)	483,606 (7)
Total^a^	1208	7,362,365	1235	7,400,737

*HFCA* health facility catchment area.

^a^27 HFCAs included in the 2022 stratification did not have strata assigned in 2019. This includes central level hospitals and health facilities that have newly opened since 2019.

## Discussion

Updating the national stratification in 2022 showed marked improvements in terms of reducing the number of highest risk strata and population at risk from when the last stratification took place in 2019. Nine districts graduated from burden reduction classification to elimination phase. Highest risk HFCAs (Stratum 4) declined by 53% whereas lowest risk HFCAs (Malaria Free) increased by 14%. Updating the stratification reduced the population at-risk by 26%. Whilst there has been great progress, some HFCAs did not improve between the 2019 and 2022 stratifications. Of 1,235 HFCAs, 169 (excluding Malaria Free) did not change strata and 12 increased strata. The absolute change in total cases in the HFCAs that increased strata was small but was enough to increase the assigned strata as CMPE seek to target any area with possible remaining transmission. For example, HC Nongma, Khammuane province increased from Malaria Free (zero total cases) in the 2019 stratification to Stratum 3 (eight total cases) in the 2022 stratification. Updating the stratification also allows CMPE to respond to changing transmission patterns and risk behaviors. For example, three of the new Strata 3 or 4 HFCAs are in Thakhek district, Khammuane province where the hypothesis drawn from case investigations was that a migrant worker at the local rubber plantation introduced malaria infection after travelling from a high burden province, either Attapeu or Savannakhet. As the levels of malaria decrease in a country and it gets nearer to elimination, it is not uncommon for small geographical and temporal shifts in transmission to become more obvious against a backdrop of the overall reduced burden^33^.

The 2022 stratification was immediately put to use by CMPE by using the updated at-risk population, reduced by 26% from 3,210,191 to 2,366,068, to recalculate case and testing numbers that need to be achieved to reach the API and annual blood examination rate (ABER) targets set out in the NSP^5^. The updated stratification also identified seven HFCAs that increased from Malaria Free or Stratum 2 to Strata 3 or 4; four HFCAs in Khammuane province and one of each in Savannakhet, Saravan and Attapeu provinces. Villages in Strata 3 and 4 HFCAs are considered for VMW allocation and five (excluding a province hospital) did not have VMWs assigned. New VMWs for villages in these HFCAs have been recruited, trained and assigned in 2022. As with all countries in the GMS, Lao PDR receives funding from the Global Fund via the RAI^34^. The 2022 stratification will be instrumental in estimating and planning commodities (e.g. LLINs and rapid diagnostic tests), training plans (for surveillance and response initiatives in burden reduction/elimination districts) and staffing (e.g. VMWs and mobile malaria workers [MMWs]) based on HFCA strata and associated targets for the next round of RAI funding (RAI4E), submission for which took place early in 2023. The advantage of having a HFCA level stratification allows more granular assessment of risk and targeting of interventions. Whilst, at district level, an area may have low enough malaria burden to apply elimination activities, there are still HFCAs as hotspots of transmission that require more intensive interventions. Table 1 in the “Methods” section presents the targeting of resources based on the results of the district and HFCA stratification.

A strength of the Lao PDR stratification is the availability and use of malaria surveillance data for all health facilities in the country. Surveillance is a core pillar of malaria control and elimination^35^ and CMPE’s investment in surveillance structures and systems facilitates data-driven processes such as stratification^36^. CMPE and UNOPS conduct biannual routine data quality assessments for all levels, as such the DHIS2 data was deemed robust enough to conduct the analysis without conducting additional field validations^37^. However, there is always the risk of underlying biases and reporting gaps that may skew reported data and misrepresent the distribution of malaria in the country. The last multiple indicator cluster survey (MICS) in Lao PDR, conducted in 2017, reported that only 43% of people with a fever seek care at a health facility^38^. Another strength of the Lao PDR stratification was to validate results against modelled case estimates from a risk map. Such comparisons hold value given predictive models can adjust for known biases such as health seeking behavior and reporting^39^. Additionally, they are able to support stratification when limited data is available to define stratums (i.e. “Unknowns”).

Several limitations to the methods of the stratification and available data were identified during the process. By documenting these limitations, they can be considered prior to the next stratification, scheduled for 2025, improving the outputs. As noted previously, 77 (6%) health facilities included in the stratification do not have recorded geographic coordinates. As such, their data could not be included in creating the risk map, modelled estimated case numbers could not be extracted from the risk map and their strata could not be validated against underlying risk. Another limitation is the extensive data cleaning work required on private sector and outbreak investigation data. As cases from these sources are aggregated to district level on DHIS2, reassigning them to closest health facility was challenging and time consuming. CMPE are already taking steps to include disaggregated data in DHIS2 so that this data is more readily available.

Whilst malaria surveillance data is readily accessible in Lao PDR, there are sources of data that have been used in stratification analyses in other countries that could be considered to further improve the stratification methodology. For example, the use of population movement data can help to disentangle local vs imported cases, which is becoming increasingly important in burden reduction districts close to the Cambodian and Vietnamese borders. Such data can be attained via travel history records^40^, through surveys or the routine surveillance systems. If available, mobile phone call records can be used to estimate movement patterns in conjunction with malaria data to identify source and sinks of infection^41^. Whilst case investigation and classification are not yet implemented for cases reported in the burden reduction districts of Lao PDR, there are plans to introduce this in the next round of guideline updates. The data on travel history and classification of local and imported cases will be of great use for locating the sources of infection. By targeting interventions at the source of malaria infection, areas with localised transmission of malaria as well as high movement to and from the locality, importation of malaria to other areas can also be reduced. A stratification analysis in Mali^8^ made use of entomological data to further tailor the stratification detailing areas with vectors susceptible to available vector control interventions. A factor of the stratification was distinguishing areas exhibiting with and without pyrethroid resistance, informing the type of LLINs each area would receive in the next distribution. Molecular and genomic surveillance have been used in low transmission settings of Thies, Senegal to detect household level transmission networks and foci clustering that support faster reactive case detection within the current surveillance framework^42^. Such tools are now being established across Tanzania, Angola and Mozambique to improve detection of resistance and diagnosis in low transmission settings^43–45^. Finally, a stratification analysis in Tanzania simulated the impact of interventions on different areas of the country through mathematical modelling^46^. The evidence generated by the mathematical modelling analysis was incorporated into the stratification to tailor intervention combinations to the varying risk profiles across the country and to ensure that interventions were not removed too soon, risking an increase in prevalence.

The increasingly focalised nature of malaria in Lao PDR means that even HFCA level may mask important differences in transmission between villages. Whilst village level data is captured in DHIS2, village level data is currently difficult to work with due to the inherent difficulties in creating and maintaining a national standardised administrative level in the health information system for all communities and villages in the country. (e.g. standardising a villages administrative status and spelling, the same village assigned to different HFCAs or to different lat/longs, missing lat/longs). The CMPE is working with MOH and the national health information system team to try and improve village level data in DHIS2, and as such a village-level stratification may be an option next time. Another consideration is whether having varying interventions for specific villages within a HFCA would be too much operational burden for already overworked health facility staff to be able to manage and record. Finally, with frequent movement between villages within HFCAs, it is fallacious to assume that malaria transmission and presence of vectors is consigned to village boundaries. Malaria foci are also often based outside of administrative villages, for example in satellite villages (known as “katos” in Lao PDR), work sites in the forest and in the fields. CMPE and WHO are working to target these sites of infection through the *P. falciparum* accelerator strategy; targeting high *P. falciparum* burden foci with intensive interventions such as targeted drug administration, intermittent preventive treatment of forest/field-goers and active fever screening^47^. The first district to receive these “accelerator” interventions was Boualapha district in Khammuane province which, as detailed in the results section, requires additional support to ensure it successfully fulfils the criteria of being an elimination district. Being able to include these non-official sites of community habitation and malaria transmission in future stratifications will make intervention planning more detailed and tailored to specific epidemiology and risk profiles.

## Conclusion

The 2022 Lao PDR malaria risk stratification shows progress in malaria control since the last stratification in 2019. The stratification also clearly identifies areas with persistent malaria transmission that need to be targeted for Lao PDR to achieve the goal of malaria elimination by 2030. Continued use of malaria surveillance data for targeting and tailoring malaria interventions is a core component of CMPE’s elimination agenda. As the number of malaria cases in Lao PDR and the international funding available decrease, stratifications will need to become more granular to target limited resources to high-risk populations. However, once elimination thresholds are reached nationwide, resources will be applied more broadly and integration into the wider health system will be vital, possibly ending the need for stratification exercises.

## Data Availability

Data available upon reasonable request to the Center of Malariology, Parasitology and Entomology, Vientiane, Lao PDR.

## Abbreviations

ABER: Annual blood examination rate
ACD: Active case detection
API: Annual parasite incidence
CHAI: Clinton Health Access Initiative
CMPE: Center of Malariology, Parasitology and Entomology
CSO: Civil Society Organisation
DHIS2: District Health Information Software 2
GMS: Greater Mekong Subregion
HC: Health center
HFCA: Health facility catchment area
IEC: Information, education, communication
IRS: Indoor residual spraying
Lao PDR: Lao People’s Democratic Republic
LLIN: Long-lasting insecticide treated net
MAP: Malaria Atlas Project
MICS: Multiple indicator cluster survey
MMW: Mobile malaria worker
NECHR: National Ethics Committee for Health Research
NSP: National Strategic Plan
PYO: Person-year-observed
RAI: Regional artemisinin-resistance initiative
TWG: Technical Working Group
UNOPS: United Nations Office for Project Services
VMW: Village malaria worker
WHO: World Health Organization
